# Longitudinal Assessment Reveals Stage‐Dependent Utility of Digital Motor Markers in SCA1

**DOI:** 10.1002/mdc3.70124

**Published:** 2025-05-07

**Authors:** Ilse H.J. Willemse, Teije van Prooije, Kirsten C.J. Kapteijns, Bart P.C. van de Warrenburg

**Affiliations:** ^1^ Department of Neurology Radboud University Medical Center, Donders Institute for Brain, Cognition and Behaviour Nijmegen The Netherlands

**Keywords:** spinocerebellar ataxia, SCA1, gait, digital biomarkers, longitudinal analysis

## Abstract

**Background:**

Clinical trials in spinocerebellar ataxias (SCA) require objective, quantifiable outcome measures sensitive to changes in disease severity.

**Objectives:**

The objective was to study the sensitivity to change of digital gait biomarkers in SCA1 over a 1‐year period.

**Methods:**

Seventeen SCA1 patients and 15 controls walked 30 seconds wearing 3 sensors at 3 walking speeds (preferred, slow, fast) at baseline and 1‐year follow‐up. Relationship between gait and clinical measures was analyzed, and standardized response means (SRMs) were calculated.

**Results:**

Toe‐off angle (TOA), variability in TOA, speed, and stride length showed significant responsiveness (SRM = −1.15, 0.61, −0.77, and −0.85). TOA, which correlated most strongly with ataxia severity and patient‐reported outcomes, demonstrated higher sensitivity to change than Scale for Assessment and Rating of Ataxia (SARA) in early‐stage disease at preferred walking speed. Gait variability measures lacked sensitivity, possibly due to the short recording period.

**Conclusions:**

Digital gait biomarkers are promising outcomes in future clinical trials in SCA1, but their sensitivity to change appears stage dependent.

Disease‐modifying interventions for spinocerebellar ataxias (SCA) are emerging.^1^ Objective and quantifiable outcome measures that can detect changes in disease severity over short periods are crucial for clinical trials that will evaluate interventions.^2^, ^3^ A growing body of literature highlights the potential of digital gait biomarkers as an outcome measure for several reasons. First, the initial presentation of cerebellar ataxia is often characterized by gait disturbances.^4^ Second, patients identify gait problems as highly disabling.^5^ Third, multiple cross‐sectional studies have shown a strong correlation between digital gait biomarkers and clinical ataxia rating scales independent of SCA subtype.^6^, ^7^, ^8^ Furthermore, recent longitudinal studies in SCA3 and SCA2 showed that digital gait measures effectively capture changes over a 1‐year period in early disease stages.^9^, ^10^

SCA1 is characterized by slowly progressive ataxia, often accompanied by extracerebellar signs like spasticity and dystonia. Compared to other repeat expansion SCAs such as SCA3, SCA1 has the fastest progression.^11^ For SCA1, cross‐sectional digital gait data need validation, and longitudinal digital gait data are lacking. We present the first, 1‐year wearable sensor gait data collected in a single‐center SCA1 cohort, aiming to identify sensitive digital gait biomarkers for future clinical trials in SCA1.

## Patients and Methods

### Subjects

As part of a longitudinal Dutch natural history and biomarker discovery study in SCA1, we included 17 symptomatic patients with SCA1 and 15 age‐ and sex‐matched healthy controls (HC). Inclusion required genetically confirmed SCA1, age ≥18 years, and the ability to walk 10 m unaided. Exclusion criteria were significant comorbidities affecting gait. This study was approved by the medical ethical committee of Arnhem‐Nijmegen (CMO‐2019‐5377). All participants provided written informed consent.

### Data Collection

All subjects underwent clinical and gait assessments at baseline and after 1 year. Gait was recorded by 3 inertial sensors (Opals by APDM Wearable Technology, Portland, USA): 2 on the feet and 1 on the lumbar spine (L5) (Supplemental Fig. S1). Data were analyzed using APDM's mobility lab software.^12^, ^13^, ^14^ Subjects walked indoors along a 10‐m path, turned, and walked back, repeating this for 30 seconds at preferred, slow, and fast walking speeds. This duration was selected to enhance test completion for as many SCA1 subjects as possible.

We adopted a hypothesis‐driven strategy, prioritizing the top 10 parameters (Table 1) from Shah et al. (2021) on gait variability in SCAs assessed by similar sensors^6^ and added speed, stride length, and lateral step variability due to the promising findings of spatial variability measures previously.^10^, ^15^, ^16^

**TABLE 1:** The characteristics, clinical outcome measures, and gait parameters in preferred walking speed on baseline and follow‐up for both SCA1 cohorts

Characteristics	Full cohort (n = 17)		Early‐disease cohort (n = 9)	
	Baseline	Follow‐up	Baseline	Follow‐up
Age (y)	49.26 ± 13.01	50.26 ± 13.01	47.99 ± 13.07	48.99 ± 13.07
Male (%)	58.82	58.82	55.55	55.55
Clinical scores				
Disease duration (y)	4.71 ± 4.10	5.71 ± 4.10	3.33 ± 3.71	4.33 ± 3.71
SARA total score	9.82 ± 3.53	12.38 ± 4.29	7.38 ± 2.71	10.33 ± 3.89
SARA posture and gait	3.65 ± 1.73	4.82 ± 1.85	2.67 ± 1.32	3.78 ± 1.79
INAS	3.35 ± 1.87	6.19 ± 1.64	2.44 ± 1.59	5.63 ± 1.51
8‐m walk test (s)	5.51 ± 0.90	5.71 ± 1.14	4.96 ± 0.60	5.10 ± 0.72
PROM‐ataxia total	–	66.53 ± 38.55	–	43.11 ± 16.14
PROM‐ataxia physical	–	45.65 ± 24.07	–	29.22 ± 10.64
FARS‐ADL	–	7.50 ± 3.67	–	5.75 ± 1.26
Gait				
Number of gait cycles	13.24 ± 2.44	13.65 ± 2.47	12.78 ± 1.56	13.11 ± 2.47
Stride length (m)	1.33 ± 0.16	1.26 ± 0.16	1.40 ± 0.11	1.34 ± 0.12
Speed (m/s)	1.28 ± 0.15	1.20 ± 0.15	1.35 ± 0.12	1.27 ± 0.14
Transverse ROM SD (degrees)	2.23 ± 0.52	2.56 ± 0.80	2.21 ± 0.50	2.62 ± 0.82
Coronal ROM SD (degrees)	0.84 ± 0.35	0.92 ± 0.34	0.70 ± 0.17	0.79 ± 0.25
Toe‐out angle SD (degrees)	3.90 ± 1.10	3.92 ± 1.12	3.31 ± 0.92	3.54 ± 0.76
Stride duration SD (s)	0.02 ± 0.006	0.03 ± 0.008	0.02 ± 0.006	0.02 ± 0.01
Toe‐off angle SD (degrees)	1.33 ± 0.32	1.54 ± 0.25	1.20 ± 0.36	1.53 ± 0.18
Toe‐off angle (degrees)	32.63 ± 3.88	29.99 ± 3.85	34.78 ± 2.63	32.67 ± 2.51
Foot strike angle SD (degrees)	1.83 ± 0.52	2.03 ± 0.48	1.68 ± 0.32	1.91 ± 0.55
Lateral step variability (cm)	6.17 ± 2.15	6.30 ± 2.32	5.42 ± 1.47	5.34 ± 1.44
Elevation midswing SD (cm)	0.62 ± 0.23	0.59 ± 0.19	0.57 ± 0.09	0.55 ± 0.16
Elevation midswing (cm)	2.62 ± 0.91	2.82 ± 0.91	2.68 ± 0.52	2.79 ± 0.53
Double support SD (%)	1.68 ± 0.52	1.88 ± 0.63	1.47 ± 0.47	1.67 ± 0.41

Abbreviations: SARA, Scale for the Assessment and Rating of Ataxia; INAS, Inventory of Non‐Ataxia Signs; PROM, patient‐reported outcome measures; FARS‐ADL, Friedreich Ataxia Rating Scale‐Activities of Daily Living; ROM, range of motion; SD, standard deviation.

We used the Scale for Assessment and Rating of Ataxia (SARA),^17^ the Inventory of Non‐Ataxia Signs (INAS),^18^ and the 8‐m walking test from the Spinocerebellar Ataxia Functional Index (SCAFI)^19^ at both baseline and follow‐up. The patient‐reported outcome measure of ataxia (PROM‐ataxia)^20^ and the Friedreich Ataxia Rating Scale‐Activities of Daily Living (FARS‐ADL)^21^ were only collected during the 1‐year follow‐up.

### Statistical Analysis

We compared gait parameters between the full group of SCA1 patients (SCA1 full cohort) and HC at baseline for all walking conditions using the Mann–Whitney U test. To control for multiple comparisons, the Benjamini‐Hochberg procedure was applied.^22^ Spearman's correlation coefficients were calculated to cross‐sectionally assess the relationship between gait parameters and clinical outcomes collected at 1‐year follow‐up. To assess differences between baseline and follow‐up in the SCA1 full cohort, we calculated the standardized response mean (SRM) and the 95% confidence interval (CI) for all gait parameters, the SARA total score $\left({\text{SARA}}_{\text{total}}\right)$, SARA posture and gait score $\;({\text{SARA}}_{p\&g}$), and the 8‐m walking test. We also calculated SRMs for a subgroup of early‐disease patients ($n=9,\;{\text{SARA}}_{\text{total}}<11$) to evaluate the potential impact of disease stage on the SRM. The cutoff of 11 was an arbitrary, convenient cohort split. Additionally, we estimated sample sizes for future clinical trials using SARA and the digital gait marker with the highest SRM as outcomes.

Toe‐off angle (TOA) emerged as a potentially sensitive outcome measure (see results). Changes in TOA may be intrinsically linked to gait ataxia specifically, or they may instead reflect changes in gait speed and stride length. To explore the relationship between these parameters, we determined Spearman's correlations for gait speed and stride length with TOA at baseline, as well as 1‐year changes in the full SCA1 cohort at preferred walking speed.

Statistical analyses were performed in R (version 4.3.1).

## Results

### Cross‐Sectional Results

**Fig. 1:**
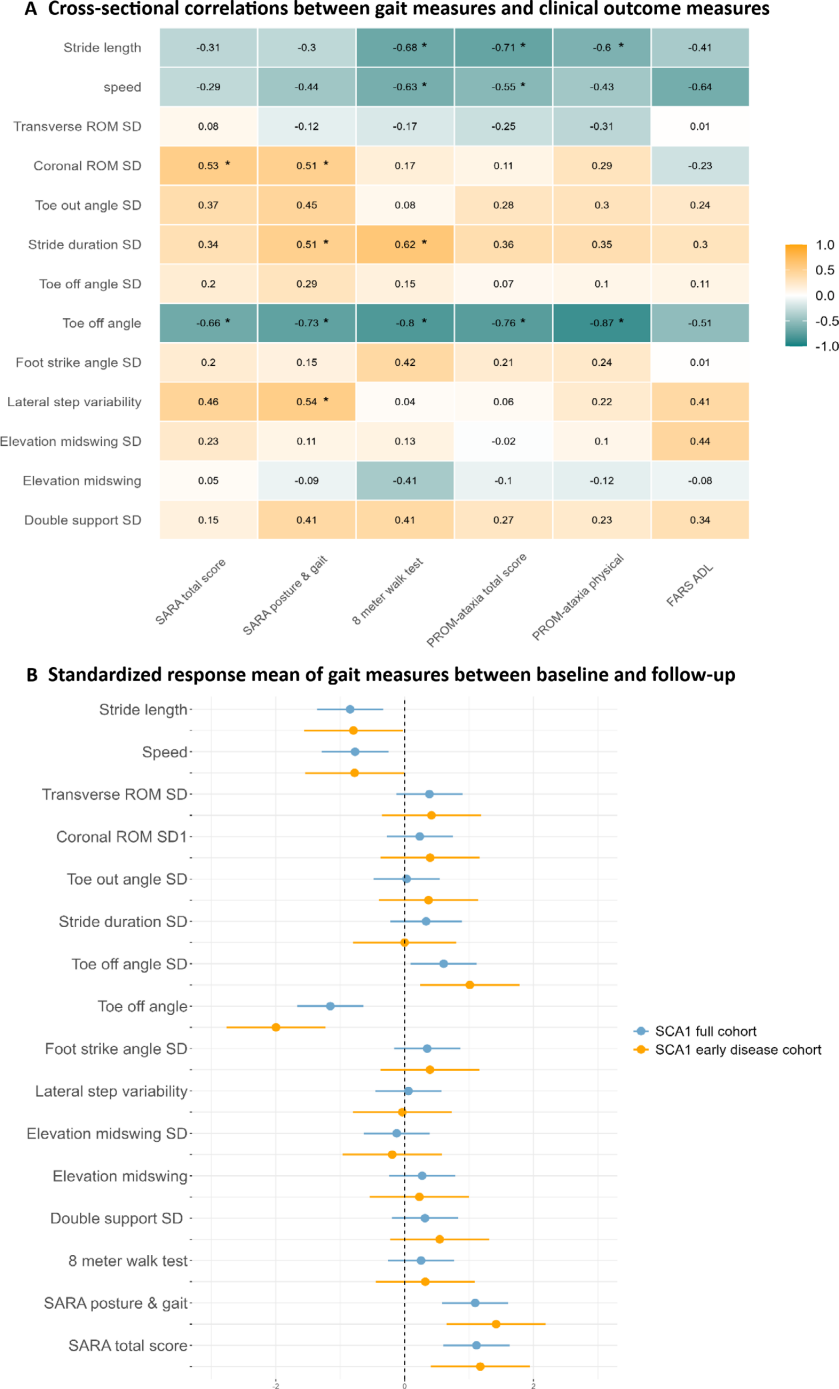
(**A**) Cross‐sectional correlations between gait measures in preferred walking speed and clinical scales in the SCA1 full cohort at follow‐up. * indicates statistical significance with *P* < 0.05. (**B**) Standardized response means of gait measures in preferred walking speed between baseline and 1‐year follow‐up in the SCA1 full cohort and SCA1 early‐disease cohort. SARA, Scale for the Assessment and Rating of Ataxia; PROM, patient‐reported outcome measures; FARS‐ADL, Friedreich Ataxia Rating Scale‐Activities of Daily Living; ROM, range of motion; SD, standard deviation.

All gait parameters significantly differed (*P* < 0.05) between the SCA1 full cohort and HC in the preferred walking speed condition at baseline. In both the slow and fast conditions, coronal range of motion standard deviation (ROM SD), stride duration SD, and foot strike angle SD showed no significant differences. Additionally, transverse ROM SD and speed did not significantly differ between groups in the slow walking speed condition (Supplemental Table S1). Cross‐sectional correlations (Fig. 1A) showed that TOA had the strongest correlation with SARA (*r* = −0.66, *P < 0.01*) and with patient‐reported outcomes measures, with the highest correlation for the physical part of the PROM‐ataxia (*r* = −0.87, *P < 0.01*) in the preferred walking speed condition. Also in the fast walking speed condition, TOA showed the highest correlation with the PROM‐ataxia physical part (*r* = −0.85, *P < 0.01*), whereas no significant correlation was found in the slow walking speed condition; no significant correlation between TOA and SARA was found in either of the two conditions.

### Longitudinal Results

Table 1 presents clinical and gait parameters at baseline and follow‐up for the SCA1 full cohort and the early‐stage subgroup at preferred walking speed. Results for the other conditions are provided in Supplemental Tables S2 and S3. After 1 year, only the gait parameters TOA, variability in TOA, speed, and stride length in the preferred walking condition significantly changed and showed statistically significant responsiveness (SRM TOA = −1.15 [95% CI: −1.67 to −0.64], SRM TOA SD = 0.61 [95% CI:0.09 to 1.12], SRM speed = −0.77 [95% CI: −1.29 to −0.25], and SRM stride length = −0.85 [95% CI: −1.36 to −0.33]); see also Figure 1B. Mean TOA, speed, and stride length decreased in SCA1, whereas the mean variability in TOA increased. In addition, we found strong baseline correlations for speed (*r* = 0.71) and stride length (*r* = 0.78) with TOA, but no correlation when considering change scores (Supplemental Tables S4 and S5). There was also a significant change in TOA, speed, and stride length in the slow and fast walking conditions (Supplemental Table S6) but not in TOA SD. In addition, there was a significant change in transverse ROM SD (SRM = −0.79 [95% CI: −1.34 to −0.23]) in the slow walking speed condition only. HC gait parameters did not significantly change (*P >* 0.05). Compared to sensitivity to change of ${\text{SARA}}_{\text{total}}$ (SRM = 1.12) and ${\text{SARA}}_{p\&g}$ (SRM = 1.10), TOA (SRM = −1.15), variability in TOA (SRM = 0.61), speed (SRM = −0.77), and stride length (SRM = −0.85) did not perform better in the preferred walking speed condition. However, in the subgroup of early‐stage SCA1, TOA (SRM = −2.0) had a higher sensitivity to change compared to ${\text{SARA}}_{\text{total}}$ (SRM = 1.17) and ${\text{SARA}}_{p\&g}$ (SRM = 1.42). Changes in TOA in the preferred walking speed condition of individual subjects in both cohorts are displayed in Supplemental Figure S2. Sample size estimation suggest that TOA requires fewer participants than SARA in early‐disease cohorts at preferred walking speed (Supplemental Fig. S3).

TOA was also the most sensitive gait measure for 1‐year progression at fast walking speed, though with a smaller effect size. At slow walking speed, stride length showed slightly better sensitivity to change than TOA, but both had smaller effect sizes compared to TOA at preferred walking speed (Supplemental Table S6).

## Discussion

This study is the first to explore longitudinal wearable sensor gait data in a single‐center SCA1 cohort to identify sensitive digital gait biomarkers for clinical trials. All examined gait parameters discriminated SCA1 patients from HC. However, after 1 year, only TOA, variability in TOA, speed, and stride length showed a statistically significant change in the preferred walking condition. TOA, which correlated most strongly with ataxia severity and patient‐reported outcomes, demonstrated higher responsiveness over a 1‐year period compared to clinical measures (eg, ${\text{SARA}}_{\text{total}}$ and ${\text{SARA}}_{p\&g}$) only in a subgroup of early‐stage SCA1 patients, especially during natural‐paced gait. Future clinical trials using TOA as an outcome measure in the preferred walking speed condition will require fewer participants than those using SARA.

In our SCA1 cohort, with a relatively rapidly progressive ataxia (+2.56 SARA points in 1 year), digital gait biomarkers did not outperform clinical scales in sensitivity to change (SRM). Previous studies where digital gait biomarkers outperformed clinical scales involved early‐stage and presymptomatic carriers of other slower‐progressing SCA genotypes^9^, ^10^ with—importantly—no significant SARA progression after 1 year. In our early‐stage SCA1 patients (+2.95 SARA points), TOA showed larger responsiveness compared to ${\text{SARA}}_{\text{total}}$ in all walking conditions, suggesting its potential in early symptomatic stages. Although the TOA outperformed ${\text{SARA}}_{p\&g}$ during the preferred walking speed condition, it did not do so in the slow walking speed condition. This was unexpected, as previous longitudinal studies showed large effects at low speeds.^9^, ^23^ Additionally, previous research suggests that maintaining balance is more challenging at lower paces.^24^

Digital gait biomarkers, particularly gait variability measures, previously showed strong correlations with ataxia severity measures.^6^, ^25^, ^26^ In our SCA1 cohort, TOA strongly correlated with SARA, but gait variability measures did not. This may be due to the cohort's mean SARA score of 12.4 or other limitations discussed below. Some gait variability measures did correlate with the ${\text{SARA}}_{p\&g}$ subscore.

A smaller TOA, previously observed in other SCAs,^6^ may be an intrinsic factor of ataxic gait or reflect a compensatory mechanism. Ataxia patients tend to take smaller steps, resulting in a reduced TOA as they adjust their gait to minimize instability.^27^, ^28^ In line with this, we found cross‐sectional correlations between TOA and gait speed and stride length, but these correlations were absent for change scores. The smaller SRM values for speed and stride length compared to TOA indicates that TOA is most sensitive to change (Supplemental Table S4), but further validation is required.

This study has some limitations. First, we used a 30‐seconds walk instead of the commonly used 2‐minutes gait assessment,^29^ enabling inclusion of more severely affected patients, but possibly limiting the reliability of gait variability measures. Second, our small cohort size, lack of presymptomatic carriers, and limited number of early‐stage SCA1 patients possibly reduced statistical power, preventing detection of changes in more gait measures. Larger longitudinal datasets across disease stages are needed to validate our findings and confirm the stage‐dependent utility of digital gait measurements in SCA1. Third, data were collected at two time points, restricting analysis of progression trends. Fourth, patient relevance of reduced TOA remains unclear despite its correlation with PROM‐ataxia. Further studies are needed to determine the predictive validity of changes in digital gait measures and to anchor these changes to outcomes that are meaningful to patients.

Sensitivity to change of the digital gait measure TOA appears to be stage dependent in this SCA1 cohort. For future clinical trials, TOA seems to be a promising surrogate endpoint for early‐stage SCA1 patients. However, due to the short recording time, the reliability regarding variability parameters in this study might be limited. Therefore, validating our findings and confirming the stage‐dependent utility of wearable sensors in SCA1 require additional efforts with larger datasets, longer recording times, and the inclusion of presymptomatic SCA1 carriers.

## Author Roles

(1) Research project: A. Conception, B. Organization, C. Execution; (2) Analysis: A. Design, B. Execution, C. Review and critique; (3) Manuscript: A. Writing of the first draft, B. Review and critique.

I.H.J.W.: 1A, 2A, 2B, 2C, 3A, 3B

T.P.: 1A, 1B, 1C, 2A, 2C, 3B

K.C.J.K.: 1A, 1B, 1C, 3B

B.P.C. van de Warrenburg: 1A, 2C, 3B

## Disclosures

**Ethical Compliance Statement:** We confirm that we have read the journal's position on issues involved in ethical publication and affirm that this work is consistent with those guidelines. This study was approved by the medical ethical committee of Arnhem‐Nijmegen (CMO‐2019‐5377). All participants provided written informed consent before enrollment.

**Funding Sources and Conflict of interest:** The study was supported by a grant from ZonMw (grant number: 404460098606). The authors have no conflicts of interest to declare.

**Financial Disclosures for the Previous 12 Months:** This project was supported by ZonMw (The Netherlands Organization for Health Research and Development; grant number: 404460098606). Bart P.C. van de Warrenburg receives research support from ZonMw, Dutch Scientific Organization, Hersenstichting, and the Christina Foundation.

## Supporting information


**Fig. S1.** Placement of 3 inertial sensors (Opals by APDM Wearable Technology‐an ERT company, Portland, OR, USA) during the gait recordings. One sensor was placed on the dorsum of each foot, and one sensor was placed at the lumbar spine at the level of L5.
**Fig. S2.** Sample size estimations for the (**A**) full cohort and (**B**) early‐disease cohort in the preferred walking speed condition with Scale for Assessment and Rating of Ataxia (SARA) and toe‐off angle as outcome measures. Calculations were performed based on effect sizes ranging from a 5% to 50% reduction.
**Fig. S3.** Spaghetti plots of the change in the digital gait outcome measure toe‐off angle between baseline and 1‐year follow‐up (Y1) for the full SCA1 cohort (green), the early‐disease cohort (blue), and healthy controls (orange). The dashed lines represent the mean change in each group.
**Table S1.** Difference in gait parameters between the SCA1 full cohort and the group of healthy controls in the preferred (blue), slow (green), and fast (yellow) walking speed condition at baseline. ROM, range of motion; SD, standard deviation.
**Table S2.** The characteristics, clinical outcome measures, and gait parameters in slow walking speed on baseline and follow‐up for both SCA1 cohorts.
**Table S3.** The characteristics, clinical outcome measures, and gait parameters in fast walking speed on baseline and follow‐up for both SCA1 cohorts.
**Table S4.** Spearman's correlations of speed and stride length with toe‐off angle on baseline.
**Table S5.** Spearman's correlations of ∆ speed and ∆ stride length with ∆ toe‐off angle over the 1‐year period.
**Table S6.** SRM result of toe‐off angle, stride length, speed, and SARA total score in all 3 conditions (preferred, fast, and slow walking speed). SRM, standardized response mean; SARA, Scale for the Assessment and Rating of Ataxia.

## Data Availability

The data that support the findings of this study are available on request from the corresponding author. The data are not publicly available due to privacy or ethical restrictions.
