# Supplementary material for: Longitudinal Assessment Reveals Stage‐Dependent Utility of Digital Motor Markers in SCA1
Source: Mov Disord Clin Pract. 2025 May 7;12(10):1622–8. doi: 10.1002/mdc3.70124 (PMC12528952; doi:10.1002/mdc3.70124)
Supplement: Supplementary file 1 — Fig. S1. Placement of 3 inertial sensors (Opals by APDM Wearable Technology‐an ERT company, Portland, OR, USA) during the gait recordings. One sensor was placed on the dorsum of each foot, and one sensor was placed at the lumbar spine at the level of L5. Fig. S2. Sample size estimations for the (A) full cohort and (B) early‐disease cohort in the preferred walking speed condition with Scale for Assessment and Rating of Ataxia (SARA) and toe‐off angle as outcome measures. Calculations were performed based on effect sizes ranging from a 5% to 50% reduction. Fig. S3. Spaghetti plots of the change in the digital gait outcome measure toe‐off angle between baseline and 1‐year follow‐up (Y1) for the full SCA1 cohort (green), the early‐disease cohort (blue), and healthy controls (orange). The dashed lines represent the mean change in each group. Table S1. Difference in gait parameters between the SCA1 full cohort and the group of healthy controls in the preferred (blue), slow (green), and fast (yellow) walking speed condition at baseline. ROM, range of motion; SD, standard deviation. Table S2. The characteristics, clinical outcome measures, and gait parameters in slow walking speed on baseline and follow‐up for both SCA1 cohorts. Table S3. The characteristics, clinical outcome measures, and gait parameters in fast walking speed on baseline and follow‐up for both SCA1 cohorts. Table S4. Spearman's correlations of speed and stride length with toe‐off angle on baseline. Table S5. Spearman's correlations of ∆ speed and ∆ stride length with ∆ toe‐off angle over the 1‐year period. Table S6. SRM result of toe‐off angle, stride length, speed, and SARA total score in all 3 conditions (preferred, fast, and slow walking speed). SRM, standardized response mean; SARA, Scale for the Assessment and Rating of Ataxia. [file MDC3-12-1622-s001.docx]

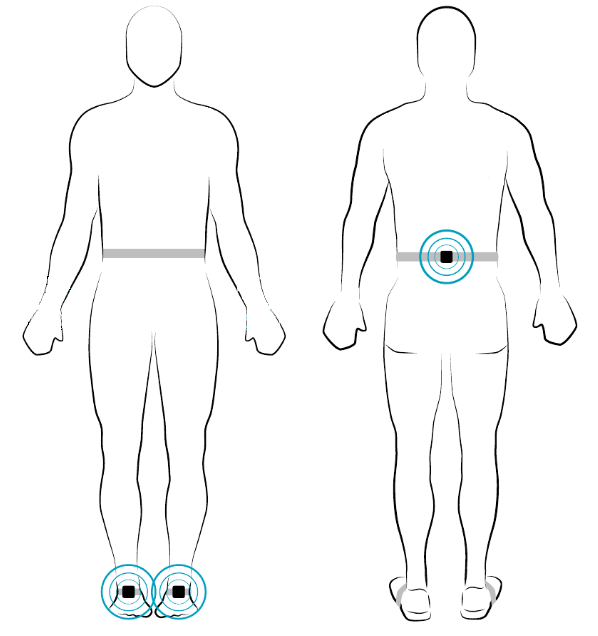


**Supplemental Figure 1:** Placement of three inertial sensors (Opals by APDM Wearable Technology-an ERT company, Portland, OR, USA) during the gait recordings. One sensor was placed on the dorsum of each foot and one sensor was placed at the lumbar spine at the level of L5.

**Supplemental Table 1:** Difference in gait parameters between the SCA1 full cohort and the group of healthy controls in the preferred (blue), slow (green) and fast (yellow) walking speed condition at baseline.

|  | SCA1 full cohort  (n = 17) | Healthy controls  (n =15 ) | Mann-Whitney U* |
| --- | --- | --- | --- |
| **Stride length (m)** | 1.33 ± 0.16 | 1.45 ± 0.09 | **0.01** |
|  | 1.13 ± 0.14 | 1.18 ± 0.06 | **0.03** |
|  | 1.46 ± 0.19 | 1.61 ± 0.12 | **0.02** |
| **Speed (m/s)** | 1.28 ± 0.15 | 1.40 ± 0.10 | **0.004** |
|  | 0.91 ± 0.14 | 0.88 ± 0.10 | 0.67 |
|  | 1.50 ± 0.19 | 1.77 ± 0.19 | **0.0006** |
| **Transverse ROM SD (degrees)** | 2.23 ± 0.52 | 1.59 ± 0.41 | **0.004** |
|  | 2.98 ± 1.20 | 2.33 ± 0.89 | 0.24 |
|  | 2.66 ± 0.60 | 1.83 ± 0.47 | **0.0006** |
| **Coronal ROM SD (degrees)** | 0.84 ± 0.35 | 0.58 ± 0.15 | **0.02** |
|  | 1.03 ± 0.52 | 0.70 ± 0.32 | 0.09 |
|  | 1.00 ± 0.38 | 0.79 ± 0.27 | 0.26 |
| **Toe out angle SD (degrees)** | 3.90 ± 1.10 | 2.42 ± 0.59 | **0.0002** |
|  | 4.02 ± 0.60 | 3.02 ± 0.85 | **0.005** |
|  | 3.32 ± 0.92 | 2.25 ± 0.52 | **0.01** |
| **Stride duration SD (s)** | 0.03 ± 0.006 | 0.02 ± 0.006 | **0.002** |
|  | 0.06 ± 0.04 | 0.05 ± 0.02 | 0.55 |
|  | 0.02 ± 0.01 | 0.02 ± 0.01 | 0.75 |
| **Toe off angle SD (degrees)** | 1.33 ± 0.32 | 1.01 ± 0.24 | **0.01** |
|  | 1.94 ± 0.60 | 1.56 ± 0.59 | **0.04** |
|  | 1.37 ± 0.46 | 1.08 ± 0.29 | **0.04** |
| **Toe off angle (degrees)** | 32.63 ± 3.88 | 36.9 ± 2.31 | **0.0004** |
|  | 29.26 ± 3.43 | 32.57 ± 3.15 | **0.01** |
|  | 34.62 ± 3.86 | 38.78 ± 2.46 | **0.001** |
| **Foot strike angle SD (degrees)** | 1.83 ± 0.52 | 1.44 ± 0.57 | **0.03** |
|  | 2.32 ± 0.81 | 2.16 ± 0.92 | 0.67 |
|  | 1.71 ± 0.74 | 1.43 ± 0.63 | 0.13 |
| **Lateral step variability (cm)** | 6.17 ± 2.15 | 3.40 ± 0.69 | **0.00001** |
|  | 5.72 ± 1.63 | 3.39 ± 1.32 | **0.0009** |
|  | 5.79 ± 1.37 | 3.84 ± 0.75 | **0.002** |
| **Elevation midswing SD (cm)** | 0.62 ± 0.23 | 0.35 ± 0.06 | **0.00001** |
|  | 0.59 ± 0.24 | 0.40 ± 0.13 | **0.008** |
|  | 0.74 ± 0.26 | 0.57 ± 0.29 | **0.01** |
| **Elevation midswing (cm)** | 2.62 ± 0.91 | 1.56 ± 0.55 | **0.0004** |
|  | 1.79 ± 0.72 | 1.21 ± 0.52 | **0.01** |
|  | 3.25 ± 1.27 | 2.20 ± 0.74 | **0.003** |
| **Double support SD (%)** | 1.68 ± 0.52 | 1.05 ± 0.23 | **0.0002** |
|  | 1.86 ± 0.62 | 1.93 ± 2.21 | **0.02** |
|  | 1.55 ± 0.44 | 1.21 ± 0.46 | **0.01** |

* To control for multiple comparisons the Benjamini-Hochberg procedure was applied.
*ROM = range of motion; SD = standard deviation***Supplemental Table 2:** The characteristics, clinical outcome measures and gait parameters in slow walking speed on baseline and follow-up for both SCA1 cohorts.

| **CHARACTERISTICS** | **Full cohort (n = 15)** | | **Early-disease cohort (n = 9)** | |
| --- | --- | --- | --- | --- |
|  | **Baseline** | **Follow-up** | **Baseline** | **Follow-up** |
| Age (years) | 47.97 ± 12.98 | 48.97 ± 12.98 | 47.99 ± 13.07 | 48.99 ± 13.07 |
| Male (%) | 53.33 | 53.33 | 55.55 | 55.55 |
| **CLINICAL SCORES** | | |  | |
| Disease duration (years) | 4.27 ± 3.99 | 5.27 ± 3.99 | 3.33 ± 3.71 | 4.33 ± 3.71 |
| SARA total score | 9.23 ± 3.16 | 12.17 ± 4.04 | 7.38 ± 2.71 | 10.33 ± 3.89 |
| SARA posture & gait | 3.33 ± 1.54 | 4.53 ± 1.78 | 2.67 ± 1.32 | 3.78 ± 1.79 |
| INAS | 3.27 ± 1.94 | 6.21 ± 1.58 | 2.44 ± 1.59 | 5.63 ± 1.51 |
| 8 meter walk test (seconds) | 5.28 ± 0.66 | 5.48 ± 0.77 | 4.96 ± 0.60 | 5.10 ± 0.72 |
| PROM-ataxia total | - | 60.67 ± 32.60 | - | 43.11 ± 16.14 |
| PROM-ataxia physical | - | 41.00 ± 20.27 | - | 29.22 ± 10.64 |
| FARS ADL | - | 6.48 ± 1.95 | - | 5.75 ± 1.26 |
| **GAIT** | | |  | |
| Number of gait cycles | 12.88 ± 1.54 | 13.44 ± 1.63 | 12.22 ± 1.20 | 13.22 ± 1.64 |
| **Stride length (m)** | 1.13 ± 0.14 | 1.00 ± 0.14 | 1.18 ± 0.06 | 1.09 ± 0.07 |
| **Speed (m/s)** | 0.91 ± 0.14 | 0.78 ± 0.14 | 0.88 ± 0.10 | 0.77 ± 0.10 |
| Transverse ROM SD (degrees) | 2.98 ± 1.20 | 2.24 ± 0.74 | 2.42 ± 0.57 | 2.09 ± 0.63 |
| Coronal ROM SD (degrees) | 1.03 ± 0.52 | 0.86 ± 0.34 | 0.87 ± 0.33 | 0.70 ± 0.17 |
| Toe out angle SD (degrees) | 4.02 ± 1.18 | 4.22 ± 1.53 | 3.59 ± 1.05 | 4.03 ± 1.69 |
| Stride duration SD  (s) | 0.06 ± 0.04 | 0.06 ± 0.03 | 0.05 ± 0.02 | 0.06 ± 0.03 |
| Toe off angle SD (degrees) | 1.94 ± 0.60 | 2.06 ± 0.52 | 1.95 ± 0.75 | 2.26 ± 0.51 |
| Toe off angle (degrees) | 29.26 ± 3.43 | 26.14 ± 2.55 | 30.48 ± 3.14 | 27.16 ± 2.70 |
| Foot strike angle SD (degrees) | 2.32 ± 0.81 | 2.57 ± 0.97 | 2.30 ± 0.93 | 2.44 ± 0.92 |
| Lateral step variability (cm) | 5.72 ± 1.63 | 4.83 ± 1.96 | 5.11 ± 1.57 | 4.48 ± 1.98 |
| Elevation midswing SD (cm) | 0.59 ± 0.24 | 0.57 ± 0.18 | 0.54 ± 0.20 | 0.54 ± 0.10 |
| Elevation midswing (cm) | 1.79 ± 0.72 | 1.92 ± 0.71 | 1.67 ± 0.51 | 1.76 ± 0.39 |
| Double support SD  (%) | 1.86 ± 0.62 | 2.11 ± 0.59 | 1.64 ± 0.62 | 1.85 ±0.54 |

*SARA = Scale for the assessment and rating of ataxia; INAS = Inventory of Non-Ataxia Signs; PROM = Patient-reported outcome measures; FARS-ADL = Friedreich Ataxia Rating Scale-Activities of Daily Living; ROM = range of motion; SD = standard deviation*

**Supplemental Table 3:** The characteristics, clinical outcome measures and gait parameters in fast walking speed on baseline and follow-up for both SCA1 cohorts.

| **CHARACTERISTICS** | **Full cohort (n = 15)** | | **Early-disease cohort (n = 9)** | |
| --- | --- | --- | --- | --- |
|  | **Baseline** | **Follow-up** | **Baseline** | **Follow-up** |
| Age (years) | 47.97 ± 12.98 | 48.97 ± 12.98 | 47.99 ± 13.07 | 48.99 ± 13.07 |
| Male (%) | 53.33 | 53.33 | 55.55 | 55.55 |
| **CLINICAL SCORES** | | |  | |
| Disease duration (years) | 4.27 ± 3.99 | 5.27 ± 3.99 | 3.33 ± 3.71 | 4.33 ± 3.71 |
| SARA total score | 9.23 ± 3.16 | 12.17 ± 4.04 | 7.38 ± 2.71 | 10.33 ± 3.89 |
| SARA posture & gait | 3.33 ± 1.54 | 4.53 ± 1.78 | 2.67 ± 1.32 | 3.78 ± 1.79 |
| INAS | 3.27 ± 1.94 | 6.21 ± 1.58 | 2.44 ± 1.59 | 5.63 ± 1.51 |
| 8 meter walk test (seconds) | 5.28 ± 0.66 | 5.48 ± 0.77 | 4.96 ± 0.60 | 5.10 ± 0.72 |
| PROM-ataxia total | - | 60.67 ± 32.60 | - | 43.11 ± 16.14 |
| PROM-ataxia physical | - | 41.00 ± 20.27 | - | 29.22 ± 10.64 |
| FARS ADL | - | 6.48 ± 1.95 | - | 5.75 ± 1.26 |
| **GAIT** | | |  | |
| Number of gait cycles | 12.38 ± 3.07 | 12.75 ± 2.11 | 12.33 ± 2.65 | 12.00 ± 2.00 |
| **Stride length (m)** | 1.46 ± 0.19 | 1.40 ± 0.18 | 1.53 ± 0.14 | 1.48 ± 0.16 |
| **Speed (m/s)** | 1.50 ± 0.19 | 1.43 ± 0.20 | 1.58 ± 0.15 | 1.53 ± 0.19 |
| Transverse ROM SD (degrees) | 2.66 ± 0.60 | 3.06 ± 0.81 | 2.86 ± 0.63 | 3.20 ± 0.69 |
| Coronal ROM SD (degrees) | 1.00 ± 0.38 | 1.03 ± 0.27 | 0.93 ± 0.26 | 0.92 ± 0.19 |
| Toe out angle SD (degrees) | 3.32 ± 0.92 | 3.40 ± 1.06 | 2.80 ± 0.62 | 2.93 ± 0.68 |
| Stride duration SD  (s) | 0.02 ± 0.01 | 0.03 ± 0.01 | 0.03 ± 0.01 | 0.03 ± 0.01 |
| Toe off angle SD (degrees) | 1.37 ± 0.46 | 1.47 ± 0.45 | 1.43 ± 0.54 | 1.49 ± 0.48 |
| Toe off angle (degrees) | 34.62 ± 3.86 | 32.55 ± 3.71 | 36.47 ± 3.28 | 34.52 ± 3.21 |
| Foot strike angle SD (degrees) | 1.71 ± 0.74 | 1.99 ± 0.91 | 1.77 ± 0.81 | 1.71 ± 0.95 |
| Lateral step variability (cm) | 5.79 ± 1.37 | 6.37 ± 2.25 | 5.21 ± 1.13 | 6.18 ± 2.29 |
| Elevation midswing SD (cm) | 0.74 ± 0.26 | 0.80 ± 0.31 | 0.81 ± 0.30 | 0.87 ± 0.33 |
| Elevation midswing (cm) | 3.25 ± 1.27 | 3.55 ± 1.21 | 3.47 ± 1.02 | 3.81 ± 1.08 |
| Double support SD  (%) | 1.55 ± 0.44 | 1.76 ± 0.62 | 1.50 ± 0.40 | 1.67 ± 0.44 |

*SARA = Scale for the assessment and rating of ataxia; INAS = Inventory of Non-Ataxia Signs; PROM = Patient-reported outcome measures; FARS-ADL = Friedreich Ataxia Rating Scale-Activities of Daily Living; ROM = range of motion; SD = standard deviation*

**Supplemental Table 4:** Spearman correlations of speed and stride length with toe-off angle on baseline.

|  | **Preferred** | |
| --- | --- | --- |
|  | **Value** | **Spearman R*** |
| **Toe-off angle (degrees)** | 32.63 ± 3.88 | - |
| **Speed (m)** | 1.28 ± 0.15 | 0.71** |
| **Stride length (m/s)** | 1.33 ± 0.16 | 0.78** |

*Correlation with Toe-off angle

**Supplemental Table 5:** Spearman correlations of ∆ speed and ∆ stride length with ∆ toe-off angle over the one year period.

|  | **Preferred** | |
| --- | --- | --- |
|  | **Value** | **Spearman R*** |
| **∆ Toe-off angle (degrees)** | -2.63 ± 2.28 | - |
| **∆ Speed (m)** | -0.08 ± 0.10 | 0.13 |
| **∆ Stride length (m/s)** | -0.07 ± 0.08 | 0.20 |

*Correlation with ∆ toe-off angle

**Supplemental Table 6:** SRM result of Toe-off angle, Stride Length, Speed and SARA total score in all three conditions (preferred, fast and slow walking speed).

|  | **Toe off angle (degrees)** | **Stride length (m)** | **Speed (m/s)** | **SARA Total score** |
| --- | --- | --- | --- | --- |
| **SRM (n = 17)**  **Preferred walking**  **Full cohort** | -1.153  (95% CI: -1.67 to -0.64) | -0.846  (95% CI: -1.36 to -0.33) | -0.769  (95% CI: -1.29 to -0.25) | 1.115  (95% CI: 0.60 to 1.63) |
| **SRM (n = 9)**  **Preferred walking**  **Early-disease** | -1.997  (95% CI: -2.8 to -1.23) | -0.795  (95% CI: -1.56 to -0.03) | -0.777  (95% CI: -1.55 to -0.01) | 1.175  (95% CI: 0.41 to 1.94) |
|  | |  |  | |
| **SRM (n = 15)**  **Slow walking**  **Full cohort** | -1.158  (95% CI: -1.71 to -0.60) | -1.115  (95% CI: -1.66 to -0.57) | -0.964  (95% CI: -1.52 to -0.41) | 1.132  (95% CI: 0.58 to 1.69) |
| **SRM (n = 9)**  **Slow walking Early-disease** | -1.362  (95% CI: -2.13 to -0.60) | -1.540  (95% CI: -2.31 to -0.77) | -1.230  (95% CI: -2.00 to -0.46) | 1.175  (95% CI: 0.41 to 1.94) |
|  | |  |  | |
| **SRM (n = 15)**  **Fast walking**  **Full cohort** | -1.336  (95% CI: -1.89 to -0.78) | -1.000  (95% CI: -1.56 to -0.44) | -0.824  (95% CI: -1.37 to -0.28) | 1.132  (95% CI: 0.58 to 1.69) |
| **SRM (n = 9)**  **Fast walking**  **Early-disease** | -1.301  (95% CI: -2.10 to -0.53) | -1.178  (95% CI: -1.96 to -0.40) | -0.812  (95% CI: -1.58 to -0.04) | 1.175  (95% CI: 0.41 to 1.94) |

*SRM = Standardized response mean; SARA = Scale for the assessment and rating of ataxia.*

**
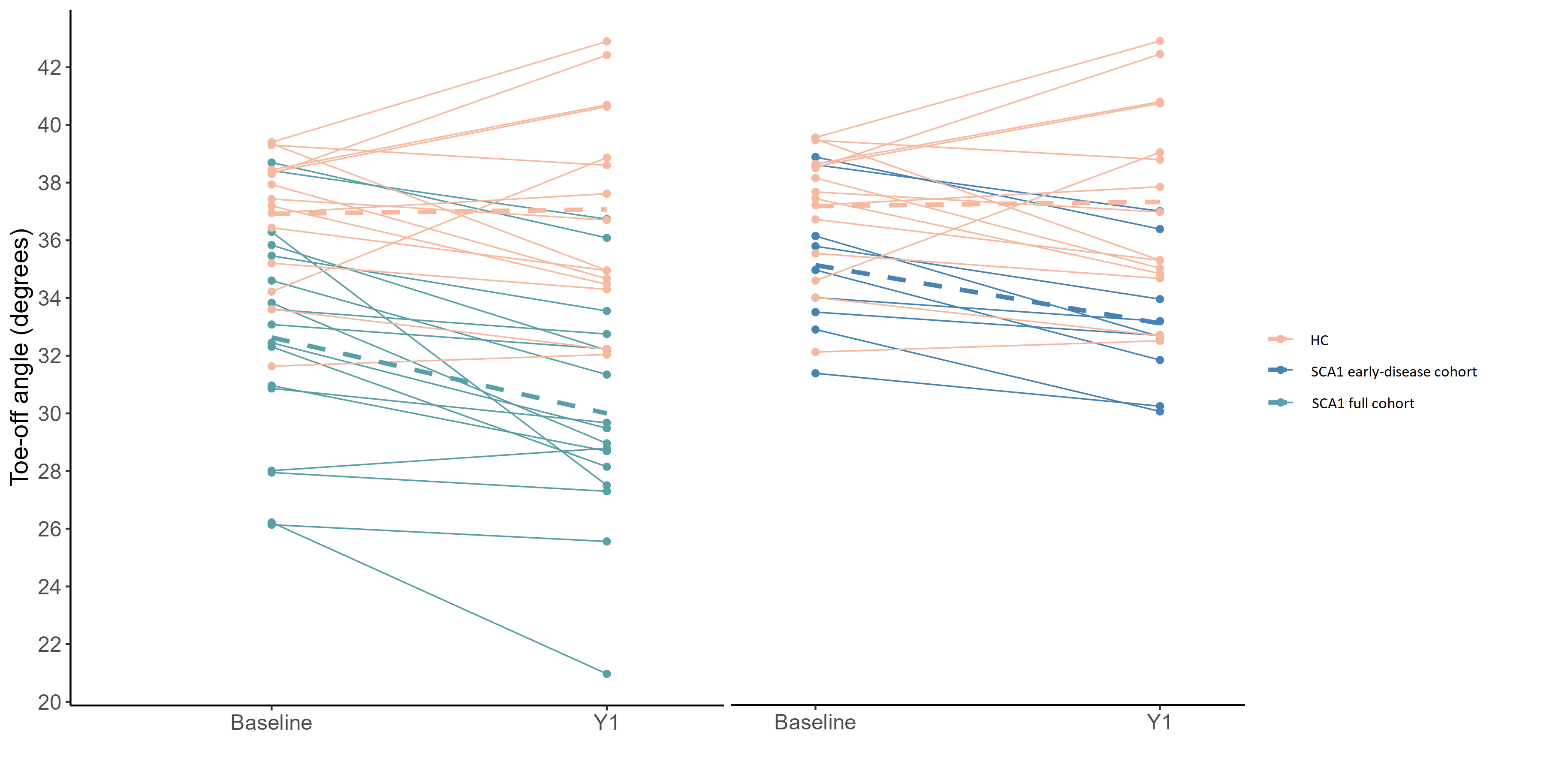
Supplemental Figure 2:** Spaghetti plots of the change in the digital gait outcome measure toe-off angle between baseline and one year follow-up (Y1) for the full SCA1 cohort (green), the early-disease cohort (blue) and healthy controls (orange). The dashed lines are representing the mean change of each group.

**
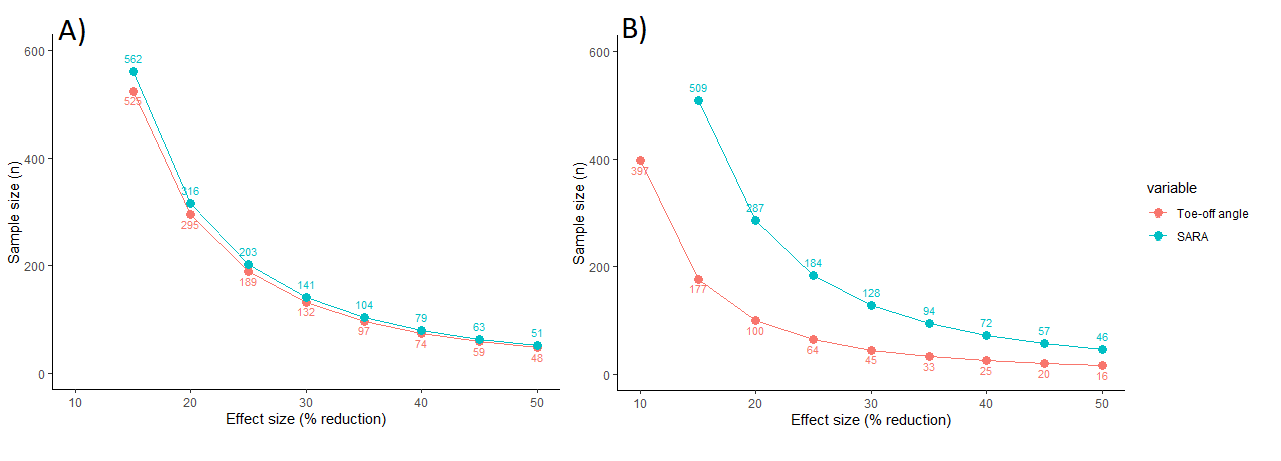
Supplemental figure 3:** Sample size estimations for the A) full cohort and B) early-disease cohort in the preferred walking speed condition with SARA and toe-off angle as outcome measures. Calculations were performed based on effect sizes ranging from a 5% to 50% reduction.
